# Suicidal incidence and gender-based discrepancies in prolonged grief disorder: insights from a meta-analysis and systematic review

**DOI:** 10.3389/fpsyt.2024.1427486

**Published:** 2024-08-15

**Authors:** Jin-Heng Tu, Yun Lu, Zong-Chao Yue, Ke-Nan Ling, Yu-Run Xing, Dan-Dan Chen, Zhi-Ren Zhu, Tian-Xi Chen

**Affiliations:** ^1^ School of Nursing and Rehabilitation, Nantong University, Nantong, China; ^2^ School of Nursing, Harbin Medical University, Harbin, Heilongjiang, China; ^3^ Department of Emergency Medicine, Affiliated Hospital of Nantong University, Nantong, Jiangsu, China

**Keywords:** prolonged grief disorder, gender differences, bereavement and mental health, suicide, systematic review and meta-analysis

## Abstract

**Background:**

In the aftermath of bereavement, our research explores the subtleties of Prolonged Grief Disorder (PGD), focusing particularly on its correlation with suicidal behaviors and their variation across genders. This study seeks to elucidate the impact of gender on these behaviors among individuals suffering from PGD, thereby enhancing our understanding and facilitating the development of tailored therapeutic interventions.

**Methods:**

By November 24th, 2023, we had rigorously reviewed key databases such as PubMed, Web of Science, Cochrane Library, PsycINFO, and Embase. Independently, two researchers conducted detailed interviews and filled out questionnaires with participants to gather demographic information and record instances of prolonged grief disorder. The study also meticulously tracked occurrences of suicidal ideation, suicide attempts, suicide deaths, and self-injury among the participants.

**Results:**

The findings indicate that 22.34% of males reported suicidal ideation (95% CI: 21.33–23.35), a figure that rises to 26.84% among females (95% CI: 25.99–27.69). Notably, 12.11% of males attempted suicide (95% CI: 11.49–12.72), marginally surpassing the 9.60% observed in females (95% CI: 9.17–10.04). More striking disparities were observed in suicide deaths, with rates for males at 3.66% (95% CI: 3.32–4.00) compared to a notably higher 7.12% for females (95% CI: 6.44–7.81). Furthermore, the incidence of self-injury was lower among males, at 2.48% (95% CI: 2.03–2.94), than in females, who reported a rate of 5.09% (95% CI: 4.69–5.49). These patterns underscore the critical need for gender-specific interventions aimed at reducing these significant disparities.

**Conclusion:**

This study distinctly underscores the profound impact of gender on the manifestation of suicidal behaviors in individuals afflicted with prolonged grief disorder. It reveals that females are more prone to suicidal ideation, self-injury, and suicide deaths, while males predominantly exhibit a higher incidence of suicide attempts and risk-taking behaviors. These unmediated trends highlight the necessity for gender-specific clinical interventions tailored to address particular behaviors and modify prevalent patterns that typically resist conventional approaches.

**Systematic review registration:**

PROSPERO (york.ac.uk), identifier CRD42023480035.

## 1 Introduction

Prolonged Grief Disorder (PGD) is a chronic condition marked by intense, enduring grief that disrupts daily life (1). Individuals with PGD often experience profound sadness and persistent yearning for the deceased, which can lead to severe functional impairments (2, 3). These impairments significantly impact their mental and emotional well-being (4). This study aims to explore the incidence of suicide among individuals with PGD, with a particular focus on gender-based differences. Understanding these differences is crucial for developing more effective, tailored interventions that address the unique challenges faced by men and women in coping with PGD.

Individuals diagnosed with Prolonged Grief Disorder (PGD) exhibit significant disruptions in daily activities, burdened by intense emotional pain, a relentless focus on memories of the deceased, and progressively deepening sensations of emptiness (5). Such profound distress and isolation often escalate to suicidal ideation and behaviors (6, 7), as those suffering may view self-harm as their sole relief from persistent sorrow. The association between PGD and suicidal tendencies encapsulates the complex emotional conflicts encountered by those impacted (8). These severe emotional states underscore the urgent need for effective interventions. Research consistently shows that gender significantly influences the grieving process and the associated risk of suicide (9, 10). Men and women employ different coping mechanisms and exhibit varying emotional responses to loss, shaped by a combination of biological factors and sociocultural expectations (11). Women, generally encouraged to express their emotions openly, benefit from enhanced social support networks (12). However, this openness can also render them more vulnerable to intense and prolonged grief, potentially leading to significant psychological distress. Conversely, societal norms often socialize men to suppress their emotions, promoting an image of stoicism and emotional control (13, 14). This suppression can result in unresolved grief, heightening the risk of adverse mental health outcomes, including depression and suicidal behaviors (15–17). Responses to grief are further influenced by factors such as hormonal changes, societal norms, and cultural practices. Hormones affect emotional regulation and stress responses, while societal norms dictate acceptable expressions of grief and coping strategies (18, 19). Cultural practices also play a significant role, as different cultures have distinct rituals and support systems that can either aid or hinder the grieving process (20, 21). Recognizing these differences is crucial for developing effective interventions. Tailored approaches that consider the unique ways in which men and women experience and cope with grief can lead to more effective treatments, ultimately reducing the incidence of suicidal behaviors among those suffering from prolonged and intense grief.

The chronic nature of Prolonged Grief Disorder (PGD), which often necessitates long-term management, requires a thorough and nuanced understanding of its extensive mental health impacts. Effective treatment models must integrate robust psychological support while accounting for gender-specific needs to comprehensively address the diverse challenges associated with grief (22, 23). This involves adopting therapeutic approaches that consider the biological, psychological, and sociocultural factors shaping grief responses. For example, interventions for women might focus on fostering emotional expression and enhancing social connectivity, while strategies for men could prioritize addressing emotional suppression and promoting healthy coping mechanisms (24–26). Furthermore, developing gender-sensitive treatment plans is critical for improving therapeutic outcomes (27). These plans should include personalized assessments to identify individual risk factors and tailor interventions accordingly. By doing so, healthcare providers can deliver more precise and effective support, ultimately enhancing the quality of care for individuals suffering from PGD. Such comprehensive approaches are essential for tackling the unique and multifaceted challenges posed by PGD. They ensure that both men and women receive appropriate care and support to navigate their grief, thereby significantly enhancing their overall mental health and well-being (28). This holistic understanding and tailored intervention framework are pivotal for achieving superior therapeutic outcomes and elevating the standard of care for those affected by this debilitating condition.

This meta-analysis employs a gender-focused lens to investigate the complex relationships between suicidal ideation, suicide attempts, suicide-related deaths, and self-injurious behaviors in individuals affected by Prolonged Grief Disorder (PGD). Our methodological approach delineates gender-specific patterns and causative factors, enhancing our comprehension of these critical issues and supporting the development of targeted prevention strategies. By integrating comprehensive demographic and clinical data, the study uncovers distinct trajectories for men and women. Furthermore, this research facilitates the creation of tailored interventions that address the unique challenges faced by both genders, thereby significantly improving therapeutic outcomes and patient care.

## 2 Methods

### 2.1 Search strategy

This review and meta-analysis adhered strictly to the PRISMA guidelines (Preferred Reporting Items for Systematic Reviews and Meta-analyses) (29). We undertook a thorough examination across major databases, including Web of Science and PubMed, with the objective of identifying studies that investigate gender disparities in suicide rates among those suffering from prolonged grief disorder, spanning from the inception of these databases until November 24, 2023. To broaden our research scope, we additionally consulted bibliographies, academic textbooks, and publications from the World Health Organization. Initially, our search was focused on uncovering links between suicide and prolonged grief disorder. We later expanded this search to incorporate both Medical Subject Heading (MeSH) terms and free text keywords such as ‘prolonged grief disorder,’ ‘complicated grief,’ ‘pathological grief,’ and ‘suicide.’ We thoroughly examined the references of all retrieved articles to identify further pertinent studies. The complete details of our search methodologies are documented in **Supplementary Table 1**.

### 2.2 Outcome indicators

Our meta-analysis meticulously examines the diverse spectrum of suicidal behaviors exhibited by those suffering from prolonged grief disorder, including suicidal ideation, suicide attempts, self-injurious behavior, and suicide deaths. Notably, we found that both suicidal ideation and attempts are key indicators of potential suicide deaths (30, 31). By broadening our perspective, we gain a more detailed understanding of the necessary preventative measures for this vulnerable population.

Suicidal ideation encompasses a state wherein individuals harbor a profound longing for death, often accompanied by recurring thoughts of self-injury or ending one’s life. Notably, these contemplations do not necessarily translate into concrete actions toward fulfilling these desires. Regarded as a precursor to actual suicide attempts, suicidal ideation can typically be identified through specific markers on mental health assessment tools, such as the ninth item on the Patient Health Questionnaire (32). Factors such as depression, a sense of hopelessness, impulsivity, and other psychiatric conditions are acknowledged as significant indicators that may lead to suicidal ideation (33, 34).

Suicide attempts represent deliberate yet non-fatal acts of self-injury. The critical juncture between contemplating and acting upon suicidal ideation hinges on the individual’s capacity to withstand the daunting fears associated with pain and mortality. This pivotal mental transition significantly informs the clinical assessment of a patient’s condition severity (35). The evaluation of these attempts is thus crucial, serving as an indicator of both the immediate danger present and the level of psychological turmoil experienced by the patient.

Suicide deaths, the fatal outcomes of self-injury actions, are unequivocally intentional. The predominant mode of such deaths involves poisoning, typically via pharmaceuticals, which is responsible for an estimated 70 to 90 percent of these incidents (36). Diverse and less common methods include asphyxiation by hanging, aquatic entrapment leading to drowning, high-impact trauma from jumping from elevated structures, self-inflicted road traffic accidents, lacerations from sharp objects, discharge of firearms, to the extremes of self-immolation (37). Data indicates that completed suicides more frequently involve these more violent methodologies (38), in contrast to the less lethal methods often associated with suicide attempts.

Self-injury, or non-suicidal self-injury (NSSI), constitutes a behavioral pattern where individuals inflict harm upon their own bodies without the intention of achieving a fatal outcome. This phenomenon is distinct from suicidal behaviors, as it does not stem from a desire to end one’s life but rather serves as a maladaptive coping mechanism to alleviate psychological distress (39). Self-injury behaviors can range from minor manifestations like superficial cuts or burns to more serious forms of harm, yet these actions are not intended to be lethal (40). Non-Suicidal Self-Injury (NSSI) is a critical clinical concern, as it reflects deep emotional distress and can escalate the risk of advancing to suicide deaths (41).

### 2.3 Study selection

In order to maintain the integrity of our collected research, we utilized Endnote software to eliminate duplicates following our search. Following rigorous selection criteria, teams of researchers independently assessed the studies, beginning with an analysis of the titles and abstracts. Acquisition of the full-text versions was contingent upon the preliminary approval of at least one reviewer.

For inclusion in our systematic review and meta-analysis, studies needed to meet the following criteria: (1) The research focused on individuals diagnosed with prolonged grief disorder. (2) The studies had to provide specific gender-based data on suicidal ideation, attempts at suicide, suicidal deaths, or self-injury, or such data could be derived from secondary sources. (3) Only observational study designs were considered.

Exclusion criteria were applied as follows: (1) Studies lacking complete data were not considered. (2) Studies published in languages other than English were excluded. (3) We also excluded studies if the full text was not accessible. (4) Studies that combined two or three outcome measures without allowing for their separate analysis were excluded. (5) In instances of participant overlap across multiple studies, the study published first was excluded to avoid data duplication.

### 2.4 Data extraction

To uphold the integrity of the data collection process, two researchers independently executed the extraction of data. In cases where discrepancies emerged, resolution was sought by consulting an external expert to maintain objectivity. We catalogued several key details from each study: the first author’s name, year of publication, location of the study, the recruitment period, the methodological design, participant ages, and the prevalence of prolonged grief disorder segregated by gender. Additionally, we noted the count of subjects exhibiting suicidal ideation, those who attempted suicide, confirmed suicide deaths, and instances of self-injury. For some studies, these figures were inferred using available rates.

### 2.5 Quality assessment

The methodological integrity of each study included in our review was meticulously assessed using the Newcastle-Ottawa Scale (NOS) devised (41). This scale scrutinizes three key aspects: the selection of study groups, the comparability between groups, and the ascertainment of exposure for case-control studies or outcome for cohort studies. It incorporates eight distinct criteria, scored on a scale from 0 to 9, where higher scores denote greater study quality. Two independent reviewers conducted the evaluations, and any disagreements were resolved through consensus. Based on the total scores, studies were categorized as low, moderate, or high quality. These classifications and detailed individual scores are documented in **Supplementary Table 2**.

### 2.6 Statistical analysis

We employed Stata 17 (version 11.0 for Windows) for all statistical analyses to ensure precise and comprehensive handling of our data. This software facilitated the quantification of gender-based discrepancies in suicidal manifestations among prolonged grief disorder patients, and we calculated odds ratios (OR) along with their 95% confidence intervals (CI) to reflect the comparative prevalence. To select the appropriate statistical model, we rigorously evaluated the level of heterogeneity among the included studies, assessing it using both the I² index and the Q test. The I² index quantified the percentage of total variation across studies due to heterogeneity rather than chance, with values of I² ≤ 40% and a P-value > 0.1 indicating low heterogeneity. For datasets meeting these criteria, we applied fixed effects models, while in cases where heterogeneity exceeded these thresholds, we used random effects models as recommended by Barili et al. to ensure the variability across studies was appropriately accounted for (42). To detect any potential publication bias, we conducted funnel plot analysis and performed Egger’s test, with the funnel plot providing a visual assessment of asymmetry and Egger’s test offering a formal statistical evaluation of potential bias. Sensitivity analyses were conducted by sequentially excluding individual studies to ensure the robustness of our findings, ensuring that no single study unduly influenced the overall results and providing confidence in the stability of our conclusions. Additionally, we performed subgroup analyses to explore potential differences based on continental origin and study design, and conducted meta-regression analyses to examine the impact of covariates such as mean age, publication year, and Newcastle-Ottawa Scale quality scores on the meta-analytical outcomes.

## 3 Results

### 3.1 Study characteristics

**Figure 1:**
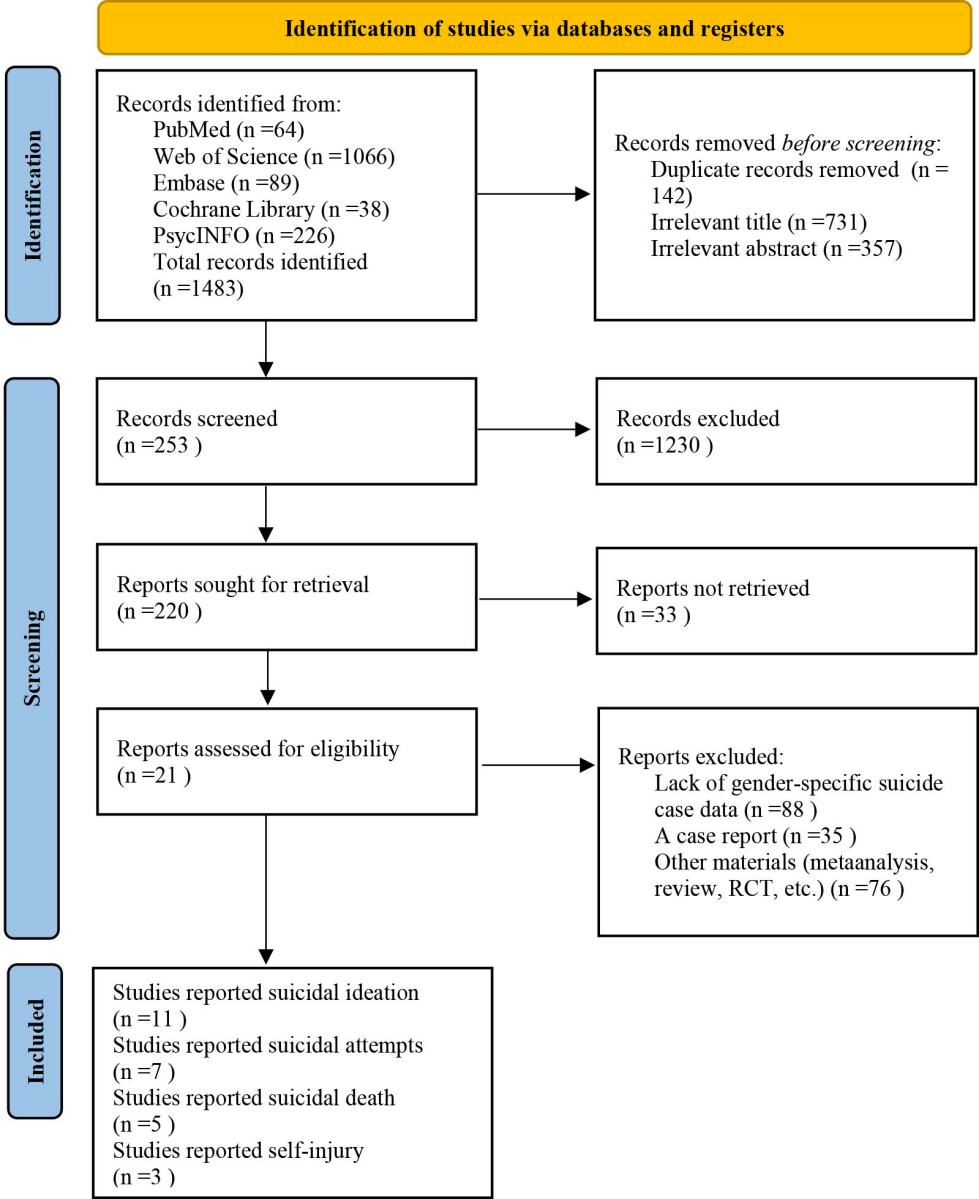
Flow chart of the study selection process.

A comprehensive search of databases yielded 1,483 potentially relevant studies. After removing 142 duplicate records, 1,341 articles remained. Of these, 731 studies were excluded due to irrelevant titles and 357 due to irrelevant abstracts, leaving 253 articles for screening. Following this, 1,230 records were excluded, narrowing the focus to 220 reports sought for retrieval. However, 33 reports were not retrieved, resulting in 21 reports assessed for eligibility. Finally, after excluding reports lacking gender-specific suicide case data, case reports, and other materials such as meta-analyses and reviews, 11 studies reported suicidal ideation, 7 reported suicidal attempts, 5 reported suicidal deaths, and 3 reported self-injury were included in the meta-analysis. Notably, some of these 21 studies included more than one type of suicidal outcome. The detailed selection process is illustrated in **Figure 1**.

**Table 1** provides a comprehensive analysis of 21 studies that assess the epidemiology and nuanced differences within prolonged grief disorder in a cohort of 83,573 patients (35,955 males and 47,618 females) from diverse global settings including the USA, UK, Sweden, South Korea, Germany, South Africa, and Norway. These studies, encompassing patient ages from 14.4 to 75.4 years, span across geographical regions with 8 based in Europe, 10 in America, 2 in Asia, and 1 in Africa. Specifically, the research delineates various manifestations of prolonged grief disorder, explores the prevalence of suicidal ideation in 11 studies, investigates suicide attempts in 7, records suicide deaths in 5, and identifies incidents of self-injury in 3 studies.

**Table 1:** Key features of the selected studies.

Author, year	Country	Period	Study design	Age	Category	M(S/N)	F(S/N)	Type of bereavements	NOS
McDonnell et al., 2022 (43)	UK	2017-2018	Cross-sectional	43.6 ± 13.0	self-injury	81/1519	455/5627	friends and family	6
Mellstrom et al., 1982 (44)	Sweden	1969-1978	Retrospective	72.0 ± 1.4	self-injury	23/2920	93/5691	spouses	8
Szanto et al., 2006 (45)	USA	2001-2004	Prospective	46.9 ± 11.3	self-injury	7/35	34/114	friends and family	8
McDonnell et al., 2022 (43)	UK	2017-2018	Cross-sectional	43.6 ± 13.0	Suicidal ideation	350/629	1551/2515	friends and family	6
Mitchell et al., 2005 (46)	USA	NR	Cross-sectional	42.7 ± 13.3	Suicidal ideation	8/35	2/15	NR	7
Song et al., 2015 (47)	South Korea	2013	Cross-sectional	46.3 ± 16.7	Suicidal ideation	47/449	39/465	friends and family	7
Szanto et al., 1997 (48)	USA	NR	Cross-sectional	65.0 ± 6.9	Suicidal ideation	16/25	23/66	family	7
Latham et al., 2004 (49)	USA	NR	Prospective	61.8 ± 13.6	Suicidal ideation	2/80	22/205	spouses	7
Grafiadeli et al., 2021 (50)	German	2020-2021	Cross-sectional	43.3 ± 13.3	Suicidal ideation	5/11	43/98	friends and family	7
Abbott et al., 2014 (51)	USA	2002-2008	Prospective	53.0 ± 13.0	Suicidal ideation	4/29	17/77	friends and family	8
Shilubane et al., 2013 (52)	South Africa	2002-2008	Cross-sectional	16.0 ± 1.7	Suicidal ideation	941/3069	978/3192	NR	7
van de Venne et al., 2020 (53)	USA	2012-2013	Cross-sectional	53.9 ± 16.3	Suicidal ideation	64/784	57/862	friends and family	8
Szanto et al., 2006 (45)	USA	2001-2004	Prospective	45.3 ± 10.7	Suicidal ideation	16/87	27/114	friends and family	8
Williams et al., 2018 (54)	USA	2001-2011	Cross-sectional	45.8 ± 10.8	Suicidal ideation	11/19	43/54	friends and family	6
Hill, 1969 (55)	UK	1905-1945	Retrospective	NR	Suicide attempts	69/538	170/945	parents	6
McDonnell et al., 2022 (43)	UK	2017-2018	Cross-sectional	43.6 ± 13.0	Suicide attempts	82/953	294/3854	friends and family	6
Wilcox et al., 2015 (56)	Sweden	2001-2004	Prospective	50.5 ± 5.9	Suicide attempts	1/261	3/276	children	8
Sibold et al., 2015 (57)	USA	2013	Cross-sectional	NR	Suicide attempts	56/4529	108/7825	NR	7
Choi et al., 2017 (58)	South Korea	2007-2013	Cross-sectional	NR	Suicide attempts	11/467	9/818	NR	7
Shilubane et al., 2013 (52)	South Africa	2002-2008	Cross-sectional	16.08 ± 1.7	Suicide attempts	1029/4949	1169/5148	NR	7
Szanto et al., 2006 (45)	USA	2001-2004	Prospective	45.3 ± 11.1	Suicide attempts	4/35	9/114	friends and family	8
Rostila et al., 2014 (59)	Sweden	1981-2002	Prospective	44.5 ± 11.5	Suicide deaths	53/357	32/217	siblings	8
Bottomley et al., 2022 (60)	USA	NR	Cross-sectional	42.3 ± 17.1	Suicide deaths	20/65	165/338	friends and family	7
Burrell et al., 2018 (61)	Norway	1992-2012	Retrospective	39.9 ± 8.5	Suicide deaths	114/6839	56/2572	parents	8
Helsing et al., 1982 (62)	USA	1963-1975	Prospective	NR	Suicide deaths	4/382	7/777	spouses	7
Rostila et al., 2013 (63)	Sweden	1981-2002	Prospective	39.8 ± 3.5	Suicide deaths	242/4618	125/1886	siblings	8

NR, not applicable; NOS, Newcastle-Ottawa scale for the assessment of the quality of non-randomized studies in meta-analyses; S/N, number of males or females with suicidal ideation or suicide attempts or suicide deaths/number of males or females with prolonged grief disorder.

Assessment of the methodological quality of the 21 studies included in our analysis revealed a promising level of rigor. None of the studies were classified as low quality. The majority, constituting 14 studies (approximately 66.67%), were deemed to be of moderate quality, while the remaining 7 studies (33.33%) achieved a high-quality rating.

### 3.2 Meta-analysis

#### 3.2.1 Suicidal ideation

Among 18,904 patients with prolonged grief disorder across eleven studies, comprising 7,488 males and 11,416 females, there was a higher observed prevalence of suicidal ideation in females, reported at 26.84% (95%CI: 25.99–27.69), compared to 22.34% in males (95%CI: 21.33–23.35). A fixed-effects model was utilized, corroborated by insignificant heterogeneity (I^2^ = 17.3%, P = 0.279). **Supplementary Figure 1A** indicates a statistically significant greater risk of suicidal ideation among females with prolonged grief disorder (OR 0.98; 95% CI 0.90–1.06; p < 0.05). The symmetry observed in the funnel plot of **Supplementary Figure 1D**, along with a non-significant result from Egger’s test (P = 0.233), suggests an absence of publication bias, and the effect sizes’ stability is further corroborated by the sensitivity analysis presented in **Supplementary Figure 1E**.

Subgroup analysis within **Supplementary Figure 1B** indicates regional variations in effect sizes: Europe displays a non-significant association (OR = 0.90, 95% CI = 0.78–1.04), America reveals a neutral effect (OR = 1.01, 95% CI = 0.78–1.31), Asia shows a non-significant increased odds (OR = 1.25, 95% CI = 0.80–1.95), and Africa presents a perfectly neutral effect (OR = 1.00, 95% CI = 0.90–1.11). The overall combined effect across regions is likewise non-significant (OR = 0.98, 95% CI = 0.90–1.06), suggesting no substantial heterogeneity amongst the studies. Subgroup analysis by study design in **Supplementary Figure 1C** shows cross-sectional studies have an almost null combined effect (OR = 0.99, 95% CI = 0.92–1.07), while prospective studies suggest a potential reduction in odds (OR = 0.58, 95% CI = 0.34–1.00), with the overall effect mirroring the global analysis (OR = 0.98, 95% CI = 0.90–1.06), denoting a consistency across different study designs.

**Supplementary Figure 1F** reveals no significant trend in effect sizes over publication years, indicating a temporal consistency in the results of the studies on the condition in question (P = 0.598). Similarly, in **Supplementary Figure 1G**, the study quality, operationalized through the Newcastle-Ottawa Scale, is shown to have a non-significant influence on the effect sizes, with a wide confidence interval signaling diverse outcomes not attributable to study quality (P = 0.429). Lastly, **Supplementary Figure 1H** illustrates that the mean age of subjects across the studies does not significantly alter the effect sizes, maintaining stability across various age groups and indicating that age demographics do not drive the observed heterogeneity in study results (P = 0.784).

#### 3.2.2 Suicide attempts

An analysis of seven studies revealed the numbers of suicide attempts among genders, including 11,732 males and 18,980 females, summing up to 30,712 patients with prolonged grief disorder. The findings indicated that the overall prevalence of suicide attempts was higher in males at 12.11% (95%CI = 11.49–12.72), compared to females at 9.60% (95%CI = 9.17–10.04). Due to the low heterogeneity (I² = 39.3%, P =0.13), a fixed-effects model was utilized. **Supplementary Figure 2A** illustrates that fixed-effect models show a statistically significant lower prevalence of suicide attempts in female patients with prolonged grief disorder (OR = 0.97, 95%CI = 0.89–1.05, P <0.05). Furthermore, the funnel plots in **Supplementary Figure 2D** and the results from Egger’s test (P = 0.574) confirmed the absence of significant publication bias in the prevalence data from the included studies. Additionally, the sensitivity analysis depicted in **Supplementary Figure 2E** demonstrated a stabilized impact on the group analyzing suicide attempts.

According to the subgroup analysis presented in **Supplementary Figure 2B**, there was a notable regional variation in effect sizes across different study locations. In Asia, there was a substantially higher odds ratio (OR = 2.14, 95% CI = 0.88–5.20), which may reflect underlying regional sociocultural factors, dietary influences, or methodological differences between studies that necessitate further investigation. In contrast, findings showed a more moderate effect in America (OR = 0.92, 95% CI = 0.67–1.26), Europe (OR = 0.91, 95% CI = 0.75–1.11), and Africa (OR = 0.98, 95% CI = 0.89–1.07), indicating little to no significant change in these regions. Overall, the pooled OR for all regions was 0.97 (95% CI = 0.89–1.05), demonstrating a consistent pattern of minimal variation across the combined regions despite the notable outlier in the Asian data. From **Supplementary Figure 2C**, subgroup analysis focusing on study design revealed little variation in the impact on results, with retrospective studies showing an (OR = 0.71, 95%CI = 0.53–0.96), prospective studies an (OR = 0.98, 95%CI = 0.34–2.86), and cross-sectional studies an (OR = 0.99, 95%CI = 0.91–1.08). Overall, the (OR = 0.97, 95%CI = 0.89–1.05), reflecting the stability and broad applicability of the study designs in this research.

The meta-regression analysis depicted through bubble charts in **Supplementary Figures 2F-H** examines various factors influencing suicide attempt rates among patients with prolonged grief disorder. In **Supplementary Figure 2H**, the analysis reveals no significant correlation between the mean age of the subjects and suicide attempt rates, as evidenced by (P=0.701). Similarly, **Supplementary Figure 2G** shows that the overall quality of the studies, assessed using the Newcastle-Ottawa Scale, does not significantly impact these rates, indicated by (P=0.726). However, **Supplementary Figure 2F** illustrates a notable but non-significant widening in gender differences regarding suicide attempts over time, marked by (P=0.439), where the publication years serve as the temporal measure. This pattern warrants a closer examination of the temporal factors that may influence such gender-related disparities in future research.

#### 3.2.3 Suicide deaths

During the examination of suicide death rates in individuals afflicted with prolonged grief disorder, data compiled from five studies involving both genders showed a total of 18,051 participants, including 12,261 males and 5,790 females. It was observed that the suicide death rate among males was lower at 3.66% (95%CI = 3.22–4.00), as opposed to females, who exhibited a rate of 7.12% (95% CI = 6.44–7.81). With minimal heterogeneity observed (I²= 13.3%, P = 0.329), the employment of a fixed-effects model was warranted. Corroborated by **Supplementary Figure 3A**, the fixed-effects model analysis demonstrated a statistically significant reduction in the prevalence of suicide deaths among male patients with prolonged grief disorder (OR = 0.77, 95% CI = 0.65–0.90, P < 0.05). Furthermore, the symmetry observed in the funnel plots depicted in **Supplementary Figure 3D**, together with the results from Egger’s test (P = 0.767), suggests the possibility of publication bias and variability among the included studies. Additionally, the sensitivity analysis presented in **Supplementary Figure 3E** confirms consistent results, even when any single study from the suicide death group is removed.

Subgroup analysis within **Supplementary Figure 3B** highlights variations in effect size by region, with Europe presenting a modest decrease in odds ratio (OR = 0.80, 95% CI = 0.68–0.95) and America demonstrating a more notable decrease (OR = 0.55, 95% CI = 0.33–0.91), leading to an overall slight reduction in the combined odds ratio across all regions (OR = 0.77, 95% CI = 0.65–0.90); conversely, examination of study design types as shown in **Supplementary Figure 3C** shows similar effect magnitudes, with prospective, cross-sectional, and retrospective analyses indicating effect estimates of 0.83 (95% CI = 0.68–1.01), 0.47 (95% CI = 0.27–0.83), and 0.76 (95% CI = 0.55–1.05) respectively, and an aggregate estimate of 0.77 (95% CI = 0.65–0.90), reinforcing the reliability of these research design approaches.

Publication years have not significantly influenced the odds ratios in prolonged grief disorder studies, as **Supplementary Figure 3F** suggests (P = 0.164), implying an overarching stability in effect sizes over time; this is further corroborated by **Supplementary Figure 3G**, where study quality, operationalized through the Newcastle-Ottawa Scale, shows no significant bearing on outcomes (P = 0.138); additionally, the mean age of subjects, according to **Supplementary Figure 3H**, does not significantly alter effect sizes (P = 0.678), indicating that the disorder’s effects are consistent across different age demographics, collectively pointing to underlying variables not captured by these analyses as potential influences on the heterogeneity observed in prolonged grief disorder research.

#### 3.2.4 Self- injury

Within the studied sample of 15,906 individuals with prolonged grief disorder, made up of 4,474 males and 11,432 females, **Supplementary Figure 4A** indicates a significantly higher prevalence of self-injury among females, at 5.09% (95% CI: 4.69–5.49%), in contrast to 2.48% among males (95% CI: 2.03–2.94%). The funnel plot in **Supplementary Figure 4B** and the sensitivity analysis in **Supplementary Figure 4C**, coupled with a non-significant Egger’s test (P = 0.742), underscore the absence of publication bias and reaffirm the stability of these findings across the studies. However, due to the limited number of studies, further meta-regression analyses and subgroup examinations could not be conducted.

## 4 Discussion

In our discussion, the meta-analytic results revealed distinct variations between genders in the prevalence of suicidal ideation, suicide attempts, suicide deaths, and incidents of self-injury in individuals suffering from prolonged grief disorder. This was established through the analysis of data collected from 21 distinct studies, which included a sample of 31,785 male participants and 47,618 female participants. It was observed that the occurrences of suicidal ideation, suicide deaths, and self-injury were more common among female subjects, whereas males exhibited a higher rate of suicide attempts.

### 4.1 Suicidal ideation

Through our meta-analytical examination, we synthesized data from 11 studies, encompassing 7,488 male and 11,416 female participants. Our findings indicate no statistically significant difference in the prevalence of suicidal ideation compared to prior reports. Unlike findings reported in a previous review, which observed a historical prevalence of 57.4%, the pooled prevalence from our analysis was notably lower, potentially due to variations in age demographics (64) Additionally, the influence of sociopsychological factors among individuals with prolonged grief disorder, such as sustained psychological distress from grief, social isolation, and role changes, might explain the variability in suicidal ideation observed across different populations. Given these findings, it is imperative that interventions for prolonged grief disorder patients are carefully designed to address these specific factors. This approach should include comprehensive sociopsychological assessments to ensure targeted support and interventions are implemented, effectively reducing the risk of suicidal ideation.

### 4.2 Suicide attempts

Within the overall population, it is observed that females have a higher risk of suicide attempts while males are more prone to suicide deaths (9, 65). Conversely, our research indicates that within the context of prolonged grief disorder, the prevalence of suicide attempts is notably higher in males (12.11%) than in females (9.60%), diverging from the typically observed patterns in general suicide epidemiology, where females usually exhibit higher attempt rates. This anomaly could be reflective of the unique stressors faced by males with prolonged grief disorder or may suggest underlying differences in the expression or processing of grief-related distress. While some scholars argue that gender may not significantly sway the likelihood of suicide attempts, being potentially influenced by a multitude of factors (66), research in diverse contexts suggests otherwise. For instance, a cross-sectional study from Brazil (67)assessed 210 individuals presenting with suicide attempts in an emergency setting, finding that a substantial majority, 68.1%, were females, often with histories of physical and gender-based abuse. In contrast, male attempters showed a predilection for psychoactive substance use. This divergence in gender-specific trends, expanding over recent decades, might also be attributed to evolving diagnostic criteria and measurement tools, potentially unearthing a higher incidence of previously unrecognized suicide attempts (68).

### 4.3 Suicide deaths

Our meta-analysis, which analyzes data from five studies encompassing 8,111 males and 5,790 females, demonstrates a significantly higher prevalence of suicide deaths in female patients suffering from prolonged grief disorder. We observed suicide death rates of 3.66% for males and 7.12% for females—rates that markedly surpass the global averages of 2.4% for males and 1.3% for females as noted by the World Health Organization (69). The results indicate that social norms, which typically discourage emotional expression and vulnerability among men (70), may lead to lower reported rates of suicide among male patients with prolonged grief disorder. This situation likely results in underreporting and subtler manifestations of suicidal behavior, which could obscure the true scale of mental health challenges faced by this group. A broader analysis, rooted in global trends of suicide mortality, is essential for comprehending the unique susceptibilities linked to prolonged grief disorder. Elevated suicide rates within this group highlight a tangled web of intense psychological pain and weakened coping abilities, exacerbated by persistent grief (71, 72). Studies emphasize that the enduring nature of grief associated with this disorder not only heightens suicide risks but also hinders recovery processes, making affected individuals highly susceptible to negative outcomes (73). This comprehensive investigation connects personal experiences with broader epidemiological trends, providing greater insight into the factors driving these increased suicide rates and underscoring the critical need for customized intervention strategies.

### 4.4 Self-injury

Statistical investigations did not identify significant differences in suicide deaths by gender among individuals with prolonged grief disorder; however, they did reveal significant trends in self-injury practices. Numerous studies indicate that women are more likely than men to engage in non-suicidal self-injury, a behavior influenced by diverse psychological, social, and biological factors that affect coping strategies across genders (74–76). Additionally, while men demonstrate higher incidences of alcohol-related self-injury, the overall occurrence of self-injurious behavior tends to be more common among women (77, 78). These results underline the intricate, gender-specific responses to grief and stress, stressing the importance of specialized support following traumatic incidents such as the loss of a spouse. Due to the lack of research in this field, the scope of further meta-regression and subpopulation analysis is limited. This also supports the importance of the further study to find detailed research and phenomenalize these factors.

### 4.5 Prevention and intervention

To effectively reduce the risk of suicide among individuals with prolonged grief disorder, a multidisciplinary approach is essential. This approach should focus on breaking down barriers to mental health care and fostering a robust support network within the community. Key measures include banning easy methods of self-destruction, such as firearms and toxic materials, which have been shown to lower suicide rates (79, 80). Policy interventions should also include community programs and educational efforts aimed at increasing mental health awareness and empowering individuals with emotional management skills. These programs should train people to recognize early signs of grief and emotional distress in themselves and others, thereby broadening their educational reach and equipping communities with resources to support those who have experienced similar mental health issues.

Gender-specific interventions are crucial: for women, increased surveillance and support through regular mental health check-ups, psychological interventions like Cognitive Behavioral Therapy (CBT) and Dialectical Behavior Therapy (DBT) to manage emotional regulation and reduce self-injury; for men, proactive preventive measures through regular screenings, and crisis intervention services tailored to men showing signs of acute distress or suicidal tendencies. Dynamic therapeutic approaches such as CBT, which helps individuals restructure negative thought patterns and develop coping mechanisms, and the Attachment, Regulation, and Competency (ARC) framework, which focuses on enhancing emotional regulation and building resilience, are effective in reducing the severity of grief and improving mental health (81, 82). Community-based programs, including educational initiatives and support networks, are essential to raise awareness about prolonged grief disorder and provide ongoing emotional and psychological support. Policy and public health campaigns are necessary to enhance mental health services, secure funding for research into effective treatments, and reduce the stigma around seeking mental health care (83–85). Developing local and global strategies that improve mental health services and ensure timely support for individuals with prolonged grief disorder is critical. Integrating these specific intervention measures and strategies into clinical practice can significantly improve the management of prolonged grief disorder and reduce the associated risk of suicide. Tailored, gender-specific approaches and supportive community networks will contribute to better outcomes for those affected by this condition.

### 4.6 Limitations

Our investigation underscores several limitations, chiefly the notable dearth of studies exploring the relationship between suicide attempts and actual suicide occurrences in individuals suffering from prolonged grief disorder, as compared to the abundance of research on suicide ideation. This gap hinders our capability to perform in-depth meta-regression or subgroup analyses that could elucidate the data’s heterogeneity in greater detail. Additionally, our analysis integrates findings from 21 studies primarily conducted in America, Europe, Africa, and Asia. This geographic concentration might not fully reflect the conditions in globally diverse or economically developing nations. Also, our analysis didn’t adjust for potential confounders, leaving some uncertainty as to whether gender disparities in suicidality are influenced by other variables. Lastly, the variation in sample sizes across the studies undoubtedly introduced a level of inconsistency to our findings.

## 5 Conclusions

This meta-analysis underscores notable differences in how prolonged grief disorder manifests between genders, noting that women tend to experience higher rates of suicidal ideation, suicide deaths, and instances of self-injury, while men are more inclined to attempt suicide. These findings emphasize the critical need for diagnostic, therapeutic, and management strategies that are specifically tailored to each gender to ensure equitable healthcare interventions. In clinical practice, these findings suggest that women require heightened surveillance, timely psychological interventions, and supportive therapies to address the higher risks of suicidal ideation and self-injury. Conversely, men may benefit from regular mental health screenings, proactive preventive measures, and crisis intervention services to manage the higher propensity for suicide attempts. Such gender-specific approaches can lead to more effective management of prolonged grief disorder, improving patient outcomes and ensuring equitable healthcare. There is a compelling need for further studies to dissect the intricate causes behind these gender-specific disparities, with the aim of developing nuanced interventions that can effectively address and mitigate these differences. Future research should focus on understanding the underlying mechanisms and testing the efficacy of gender-tailored interventions in real-world clinical settings.

## Data Availability

The original contributions presented in the study are included in the article/**Supplementary Material**. Further inquiries can be directed to the corresponding author.

## Supplementary material

Supplementary Figure 1 Meta-analysis of suicidal ideation: **(A)** Forest plot displaying the combined odds ratios; **(B)** Forest plot of combined odds ratios (analyzed by continent); **(C)** Forest plot of combined odds ratios (analyzed by study design); **(D)** Funnel plot to evaluate potential publication biases; **(E)** Chart for sensitivity analysis of the included studies; **(F)** Meta-regression analysis chart based on the publication year; **(G)** Meta-regression analysis chart according to the Newcastle-Ottawa Scale quality scores; **(H)** Meta-regression analysis chart for the mean age of subjects.

Supplementary Figure 2 Meta-analysis of Suicide attempts: **(A)** Forest plot displaying the combined odds ratios; **(B)** Forest plot of combined odds ratios (analyzed by continent); **(C)** Forest plot of combined odds ratios (analyzed by study design); **(D)** Funnel plot to evaluate potential publication biases; **(E)** Chart for sensitivity analysis of the included studies; **(F)** Meta-regression analysis chart based on the publication year; **(G)** Meta-regression analysis chart according to the Newcastle-Ottawa Scale quality scores; **(H)** Meta-regression analysis chart for the mean age of subjects.

Supplementary Figure 3 Meta-analysis of suicide deaths: **(A)** Forest plot displaying the combined odds ratios; **(B)** Forest plot of combined odds ratios (analyzed by continent); **(C)** Forest plot of combined odds ratios (analyzed by study design); **(D)** Funnel plot to evaluate potential publication biases; **(E)** Chart for sensitivity analysis of the included studies; **(F)** Meta-regression analysis chart based on the publication year; **(G)** Meta-regression analysis chart according to the Newcastle-Ottawa Scale quality scores; **(H)** Meta-regression analysis chart for the mean age of subjects.

Supplementary Figure 4 Meta-analysis of self-injury: **(A)** Forest plot displaying the combined odds ratios; **(B)** Funnel plot to evaluate potential publication biases; **(C)** Chart for sensitivity analysis of the included studies.

## References

[1] Eisma MC . Prolonged grief disorder in ICD-11 and DSM-5-TR: Challenges and controversies. Aust N Z J Psychiatry. (2023) 57:944–51. doi: 10.1177/00048674231154206 PMC10291380 36748103

[2] Duffy M Wild J . Living with loss: a cognitive approach to prolonged grief disorder - incorporating complicated, enduring and traumatic grief. Behav Cognit Psychother. (2023) 51:645–58. doi: 10.1017/S1352465822000674 37170765

[3] Donaldson ZR Shear MK . Neurobiology and treatment advances for prolonged grief disorder. Neuropsychopharmacology. (2024) 49:309–10. doi: 10.1038/s41386-023-01663-8 PMC10700485 37479860

[4] Szuhany KL Malgaroli M Miron CD Simon NM . Prolonged grief disorder: course, diagnosis, assessment, and treatment. Focus (Am Psychiatr Publ). (2021) 19:161–72. doi: 10.1176/appi.focus.20200052 PMC8475918 34690579

[5] Prigerson HG Boelen PA Xu J Smith KV Maciejewski PK . Validation of the new DSM-5-TR criteria for prolonged grief disorder and the PG-13-Revised (PG-13-R) scale. World Psychiatry. (2021) 20:96–106. doi: 10.1002/wps.20823 33432758 PMC7801836

[6] Harris CB Brookman R O'Connor M . It's not who you lose, it's who you are: Identity and symptom trajectory in prolonged grief. Curr Psychol. (2023) 42:11223–33. doi: 10.1007/s12144-021-02343-w PMC8536249 34720547

[7] Werner K Wick JY . Bereavement and loss: understanding grief in older people. Sr Care Pharm. (2024) 39:98–104. doi: 10.4140/TCP.n.2024.98 38379138

[8] Na PJ Fischer IC Shear KM Pietrzak RH . Prevalence, correlates, and psychiatric burden of prolonged grief disorder in U.S. Military veterans: results from a nationally representative study. Am J Geriatr Psychiatry. (2023) 31:543–8. doi: 10.1016/j.jagp.2023.02.007 PMC11143540 36878740

[9] Carretta RF McKee SA Rhee TG . Gender differences in risks of suicide and suicidal behaviors in the USA: A narrative review. Curr Psychiatry Rep. (2023) 25:809–24. doi: 10.1007/s11920-023-01473-1 PMC11225381 37930559

[10] Maccallum F Lundorff M Johannsen M Farver-Vestergaard I O'Connor M . An exploration of gender and prolonged grief symptoms using network analysis. Psychol Med. (2023) 53:1770–7. doi: 10.1017/S0033291721003391 34503594

[11] Graves BS Hall ME Dias-Karch C Haischer MH Apter C . Gender differences in perceived stress and coping among college students. PloS One. (2021) 16:e0255634. doi: 10.1371/journal.pone.0255634 34383790 PMC8360537

[12] Gariépy G Honkaniemi H Quesnel-Vallée A . Social support and protection from depression: systematic review of current findings in Western countries. Br J Psychiatry. (2016) 209:284–93. doi: 10.1192/bjp.bp.115.169094 27445355

[13] King TL Shields M Sojo V Daraganova G Currier D O'Neil A . Expressions of masculinity and associations with suicidal ideation among young males. BMC Psychiatry. (2020) 20:228. doi: 10.1186/s12888-020-2475-y 32398056 PMC7218581

[14] Herreen D Rice S Currier D Schlichthorst M Zajac I . Associations between conformity to masculine norms and depression: age effects from a population study of Australian men. BMC Psychol. (2021) 9:32. doi: 10.1186/s40359-021-00533-6 33608063 PMC7893732

[15] Peña-Vargas C Armaiz-Peña G Castro-Figueroa E . A biopsychosocial approach to grief, depression, and the role of emotional regulation. Behav Sci (Basel). (2021) 11:123–34. doi: 10.3390/bs11080110 PMC8389251 34436100

[16] O'Gorman KM Wilson MJ Seidler ZE English D Zajac IT Fisher KS . Male-type depression mediates the relationship between avoidant coping and suicidal ideation in men. Int J Environ Res Public Health. (2022) 19:875–90. doi: 10.3390/ijerph191710874 PMC9517898 36078589

[17] Gupta S Fischer J Roy S Bhattacharyya A . Emotional regulation and suicidal ideation-Mediating roles of perceived social support and avoidant coping. Front Psychol. (2024) 15:1377355. doi: 10.3389/fpsyg.2024.1377355 38629033 PMC11018903

[18] Recksiedler C Loter K Klaas HS Hollstein B Perrig-Chiello P . Social dimensions of personal growth following widowhood: A three-wave study. Gerontology. (2018) 64:344–60. doi: 10.1159/000485916 29402839

[19] Seiler A von Känel R Slavich GM . The psychobiology of bereavement and health: A conceptual review from the perspective of social signal transduction theory of depression. Front Psychiatry. (2020) 11:565239. doi: 10.3389/fpsyt.2020.565239 33343412 PMC7744468

[20] Ayebare E Lavender T Mweteise J Nabisere A Nendela A Mukhwana R . The impact of cultural beliefs and practices on parents' experiences of bereavement following stillbirth: a qualitative study in Uganda and Kenya. BMC Pregnancy Childbirth. (2021) 21:443. doi: 10.1186/s12884-021-03912-4 34172018 PMC8228937

[21] Zorlu S Memis A Yumusak M . Religious and cultural practices of muslims living in central anatolia on death and mourning: A qualitative study from Turkey. J Relig Health. (2022) 61:4934–58. doi: 10.1007/s10943-022-01607-4 PMC9299408 35859074

[22] Chen Z Ying J Ingles J Zhang D Rajbhandari-Thapa J Wang R . Gender differential impact of bereavement on health outcomes: evidence from the China Health and Retirement Longitudinal Study 2011-2015. BMC Psychiatry. (2020) 20:514. doi: 10.1186/s12888-020-02916-2 33092555 PMC7583229

[23] Abdul Samad FD Pereira XV Chong SK Abdul Latif MHB . Interpersonal psychotherapy for traumatic grief following a loss due to COVID-19: a case report. Front Psychiatry. (2023) 14:1218715. doi: 10.3389/fpsyt.2023.1218715 37840803 PMC10576431

[24] Stelzer EM Atkinson C O'Connor MF Croft A . Gender differences in grief narrative construction: a myth or reality? Eur J Psychotraumatol. (2019) 10:1688130. doi: 10.1080/20008198.2019.1688130 31807234 PMC6882469

[25] Yeung NC Chow TS . Coping with my own way: Mediating roles of emotional expression and social support seeking in the associations between individual differences and posttraumatic growth. Health Psychol Open. (2019) 6:2055102919846596. doi: 10.1177/2055102919846596 31105967 PMC6503603

[26] Logan N Krysinska K Andriessen K . Impacts of suicide bereavement on men: a systematic review. Front Public Health. (2024) 12:1372974. doi: 10.3389/fpubh.2024.1372974 38655522 PMC11035897

[27] Heidari S Babor TF Castro P Tort S Curno M . [Sex and Gender Equity in Research: rationale for the SAGER guidelines and recommended use]. Epidemiol Serv Saude. (2017) 26:665–75. doi: 10.5123/S1679-49742017000300025 28443945

[28] Temmerman M Khosla R Laski L Mathews Z Say L . Women's health priorities and interventions. Bmj. (2015) 351:h4147. doi: 10.1136/bmj.h4147 26371215

[29] Page MJ McKenzie JE Bossuyt PM Boutron I Hoffmann TC Mulrow CD . The PRISMA 2020 statement: an updated guideline for reporting systematic reviews. Bmj. (2021) 372:n71. doi: 10.1136/bmj.n71 33782057 PMC8005924

[30] Nock MK Borges G Bromet EJ Cha CB Kessler RC Lee S . Suicide and suicidal behavior. Epidemiol Rev. (2008) 30:133–54. doi: 10.1093/epirev/mxn002 PMC2576496 18653727

[31] Wisnousky H Lazzara N Ciarletta M Pelton M Chinchilli VM Ssentongo AE . Rates and risk factors for suicidal ideation, suicide attempts and suicide deaths in persons with HIV: a protocol for a systematic review and meta-analysis. BMJ Open. (2021) 11:e037154. doi: 10.1136/bmjopen-2020-037154 PMC7925913 33550223

[32] Kim S Lee HK Lee K . Which PHQ-9 items can effectively screen for suicide? Machine learning approaches. Int J Environ Res Public Health. (2021) 18:3339–50. doi: 10.3390/ijerph18073339 PMC8036742 33804879

[33] Ribeiro JD Huang X Fox KR Franklin JC . Depression and hopelessness as risk factors for suicide ideation, attempts and death: meta-analysis of longitudinal studies. Br J Psychiatry. (2018) 212:279–86. doi: 10.1192/bjp.2018.27 29587888

[34] Riera-Serra P Navarra-Ventura G Castro A Gili M Salazar-Cedillo A Ricci-Cabello I . Clinical predictors of suicidal ideation, suicide attempts and suicide death in depressive disorder: a systematic review and meta-analysis. Eur Arch Psychiatry Clin Neurosci. (2023) 273:1081–99. doi: 10.1007/s00406-023-01716-5 PMC11422269 38015265

[35] Nathan R Bhandari S . Risk assessment in clinical practice: a framework for decision-making in real-world complex systems. BJPsych Adv. (2024) 30:53–63. doi: 10.1192/bja.2022.67

[36] Hawton K Simkin S Deeks J . Co-proxamol and suicide: A study of national mortality statistics and local non-fatal self-poisonings. BMJ: Br Med J. (2003) 326:1006–8. doi: 10.1136/bmj.326.7397.1006 PMC154756 12742920

[37] Wu KC Chen YY Yip PS . Suicide methods in Asia: implications in suicide prevention. Int J Environ Res Public Health. (2012) 9:1135–58. doi: 10.3390/ijerph9041135 PMC3366604 22690187

[38] Zhu RT Ma ZY Jia CX Zhou L . Completed suicide with violent and non-violent methods by the elderly in Rural China: A psychological autopsy study. Front Psychiatry. (2021) 12:624398. doi: 10.3389/fpsyt.2021.624398 34211408 PMC8239144

[39] Heath NL Carsley D De Riggi ME Mills D Mettler J . The relationship between mindfulness, depressive symptoms, and non-suicidal self-injury amongst adolescents. Arch Suicide Res. (2016) 20:635–49. doi: 10.1080/13811118.2016.1162243 26984524

[40] Zetterqvist M . The DSM-5 diagnosis of nonsuicidal self-injury disorder: a review of the empirical literature. Child Adolesc Psychiatry Ment Health. (2015) 9:31. doi: 10.1186/s13034-015-0062-7 26417387 PMC4584484

[41] Poudel A Lamichhane A Magar KR Khanal GP . Non suicidal self-injury and suicidal behavior among adolescents: co-occurrence and associated risk factors. BMC Psychiatry. (2022) 22:96. doi: 10.1186/s12888-022-03763-z 35139825 PMC8827284

[42] Barili F Parolari A Kappetein PA Freemantle N . Statistical Primer: heterogeneity, random- or fixed-effects model analyses? Interact Cardiovasc Thorac Surg. (2018) 27:317–21. doi: 10.1093/icvts/ivy163 29868857

[43] McDonnell S Flynn S Shaw J Smith S McGale B Hunt IM . Suicide bereavement in the UK: Descriptive findings from a national survey. Suicide Life Threat Behav. (2022) 52(5):887–97. doi: 10.1111/sltb.12874 PMC9790485 35611626

[44] Mellström D Nilsson A Odén A Rundgren A Svanborg A . Mortality among the widowed in Sweden. Scand J Soc Med. (1982) 10(2):33–41. doi: 10.1177/140349488201000201 7178869

[45] Szanto K Shear MK Houck PR Reynolds CF 3rd Frank E Caroff K . Indirect self-destructive behavior and overt suicidality in patients with complicated grief. J Clin Psychiatry. (2006) 67(2):233–9. doi: 10.4088/jcp.v67n0209 16566618

[46] Mitchell AM Kim Y Prigerson HG Mortimer MK . Complicated grief and suicidal ideation in adult survivors of suicide. Suicide Life Threat Behav. (2005) 35(5):498–506. doi: 10.1521/suli.2005.35.5.498 16268767

[47] Song IH Kwon SW Kim JE . Association between suicidal ideation and exposure to suicide in social relationships among family, friend, and acquaintance survivors in South Korea. Suicide Life Threat Behav. (2015) 45(3):376–90. doi: 10.1111/sltb.12158 25845314

[48] Szanto K Prigerson H Houck P Ehrenpreis L Reynolds CF 3rd . Suicidal ideation in elderly bereaved: the role of complicated grief. Suicide Life Threat Behav. (1997) 27(2):194–207. 9260302

[49] Latham AE Prigerson HG . Suicidality and bereavement: complicated grief as psychiatric disorder presenting greatest risk for suicidality. Suicide Life Threat Behav. (2004) 34(4):350–62. doi: 10.1521/suli.34.4.350.53737 PMC1459278 15585457

[50] Grafiadeli R Glaesmer H Hofmann L Schäfer T Wagner B . Suicide risk after suicide bereavement: The role of loss-related characteristics, mental health, and hopelessness. J Psychiatr Res. (2021) 144:184–9. doi: 10.1016/j.jpsychires.2021.09.056 34673315

[51] Abbott CH Prigerson HG Maciejewski PK . The influence of patients' quality of life at the end of life on bereaved caregivers' suicidal ideation. J Pain Symptom Manage. (2014) 48(3):459–64. doi: 10.1016/j.jpainsymman.2013.09.011 PMC4048331 24321508

[52] Shilubane HN Ruiter RA van den Borne B Sewpaul R James S Reddy PS . Suicide and related health risk behaviours among school learners in South Africa: results from the 2002 and 2008 national youth risk behaviour surveys. BMC Public Health. (2013) 13:926. doi: 10.1186/1471-2458-13-926 24093214 PMC3851142

[53] van de Venne J Cerel J Moore M Maple M . Sex Differences in Mental Health Outcomes of Suicide Exposure. Arch Suicide Res. (2020) 24(2):158–85. doi: 10.1080/13811118.2019.1612800 31081470

[54] Williams JL Eddinger JR Rynearson EK Rheingold AA . Prevalence and Correlates of Suicidal Ideation in a Treatment-Seeking Sample of Violent Loss Survivors. Crisis. (2018) 39(5):377–85. doi: 10.1027/0227-5910/a000520 29848082

[55] Hill OW . The association of childhood bereavement with suicidal attempt in depressive illness. Br J Psychiatry. (1969) 115(520):301–4. doi: 10.1192/bjp.115.520.301 5794123

[56] Wilcox HC Mittendorfer-Rutz E Kjeldgård L Alexanderson K Runeson B . Functional impairment due to bereavement after the death of adolescent or young adult offspring in a national population study of 1,051,515 parents. Soc Psychiatry Psychiatr Epidemiol. (2015) 50(8):1249–56. doi: 10.1007/s00127-014-0997-7 25552253

[57] Sibold J Edwards E Murray-Close D Hudziak JJ . Physical activity, sadness, and suicidality in bullied US adolescents. J Am Acad Child Adolesc Psychiatry. (2015) 54(10):808–15. doi: 10.1016/j.jaac.2015.06.019 26407490

[58] Choi J Lee M Ki M Lee JY Song YJ Kim M . Risk factors for feelings of sadness and suicide attempts among cancer survivors in South Korea: findings from nationwide cross-sectional study (KNHANES IV-VI). BMJ Open. (2017) 7(12):e016130. doi: 10.1136/bmjopen-2017-016130 PMC5735398 29247081

[59] Rostila M Saarela J Kawachi I . "The psychological skeleton in the closet": mortality after a sibling's suicide. Soc Psychiatry Psychiatr Epidemiol. (2014) 49(6):919–27. doi: 10.1007/s00127-013-0780-1 24126558

[60] Bottomley JS Feigelman WT Rheingold AA . Exploring the mental health correlates of overdose loss. Stress Health. (2022) 38(2):350–63. doi: 10.1002/smi.3092 PMC11267624 34448352

[61] Burrell LV Mehlum L Qin P . Sudden parental death from external causes and risk of suicide in the bereaved offspring: A national study. J Psychiatr Res. (2018) 96:49–56. doi: 10.1016/j.jpsychires.2017.09.023 28965005

[62] Helsing KJ Comstock GW Szklo M . Causes of death in a widowed population. Am J Epidemiol. (1982) 116(3):524–32. doi: 10.1093/oxfordjournals.aje.a113436 7124718

[63] Rostila M Saarela J Kawachi I . Suicide following the death of a sibling: a nationwide follow-up study from Sweden. BMJ Open. (2013) 3(4):e002618. doi: 10.1136/bmjopen-2013-002618 PMC3641510 23624991

[64] Molina N Viola M Rogers M Ouyang D Gang J Derry H . Suicidal ideation in bereavement: A systematic review. Behav Sci (Basel). (2019) 9:253–67. doi: 10.3390/bs9050053 PMC6562884 31091772

[65] Sterne J Egger M . Regression methods to detect publication and other bias in meta-analysis. Publ Bias Meta-Analysis: Prevention Assess Adjustments. (2006) 19:99–110. doi: 10.1002/0470870168.ch6

[66] Nicolini ME Gastmans C Kim SYH . Psychiatric euthanasia, suicide and the role of gender. Br J Psychiatry. (2022) 220:10–3. doi: 10.1192/bjp.2021.95 PMC8777112 35045892

[67] Stefanello S Cais CF Mauro ML Freitas GV Botega NJ . Gender differences in suicide attempts: preliminary results of the multisite intervention study on suicidal behavior (SUPRE-MISS) from Campinas, Brazil. Braz J Psychiatry. (2008) 30:139–43. doi: 10.1590/S1516-44462006005000063 18176725

[68] Freeman A Mergl R Kohls E Székely A Gusmao R Arensman E . A cross-national study on gender differences in suicide intent. BMC Psychiatry. (2017) 17:234. doi: 10.1186/s12888-017-1398-8 28662694 PMC5492308

[69] World Health Organization . Suicide in the world: global health estimates. Geneva:World Health Organization (2019). Available at: https://www.who.int/publications/i/item/9789240026643

[70] Croft A Atkinson C May AM . Promoting gender equality by supporting men’s emotional flexibility. Policy Insights Behav Brain Sci. (2021) 8:42–9. doi: 10.1177/2372732220984491

[71] Mitchell AM Kim Y Prigerson HG Mortimer-Stephens M . Complicated grief in survivors of suicide. Crisis. (2004) 25:12–8. doi: 10.1027/0227-5910.25.1.12 15384652

[72] Prigerson HG Horowitz MJ Jacobs SC Parkes CM Aslan M Goodkin K . Prolonged grief disorder: Psychometric validation of criteria proposed for DSM-V and ICD-11. PloS Med. (2009) 6:e1000121. doi: 10.1371/journal.pmed.1000121 19652695 PMC2711304

[73] Levi-Belz Y Ben-Yaish T . Prolonged grief symptoms among suicide-loss survivors: the contribution of intrapersonal and interpersonal characteristics. Int J Environ Res Public Health. (2022) 19:10545–60. doi: 10.3390/ijerph191710545 PMC9518413 36078261

[74] Cipriano A Cella S Cotrufo P . Nonsuicidal self-injury: A systematic review. Front Psychol. (2017) 8:1946. doi: 10.3389/fpsyg.2017.01946 29167651 PMC5682335

[75] Victor SE Muehlenkamp JJ Hayes NA Lengel GJ Styer DM Washburn JJ . Characterizing gender differences in nonsuicidal self-injury: Evidence from a large clinical sample of adolescents and adults. Compr Psychiatry. (2018) 82:53–60. doi: 10.1016/j.comppsych.2018.01.009 29407359 PMC5845831

[76] Victor SE Hipwell AE Stepp SD Scott LN . Parent and peer relationships as longitudinal predictors of adolescent non-suicidal self-injury onset. Child Adolesc Psychiatry Ment Health. (2019) 13:1. doi: 10.1186/s13034-018-0261-0 30622642 PMC6317237

[77] Brooks F Chester KL Klemera E Magnusson J . Intentional self-harm in adolescence: An analysis of data from the Health Behavior in School-aged Children (HBSC) survey for England 2014. Public Health England. Report number: PHE publications gateway number: 2017069. (2017), 1–61. Available at: https://dera.ioe.ac.uk/id/eprint/29451/

[78] Lange S Roerecke M Orpana H Bagge C Rehm J . Alcohol use and the gender-specific risk of suicidal behavior: a systematic review and meta-analysis protocol. Syst Rev. (2022) 11:279. doi: 10.1186/s13643-022-02159-0 36564843 PMC9783973

[79] Rodríguez Andrés A Hempstead K . Gun control and suicide: the impact of state firearm regulations in the United States 1995-2004. Health Policy. (2011) 101:95–103. doi: 10.1016/j.healthpol.2010.10.005 21044804

[80] Langmann C . Effect of firearms legislation on suicide and homicide in Canada from 1981 to 2016. PloS One. (2020) 15:e0234457. doi: 10.1371/journal.pone.0234457 32555647 PMC7302582

[81] Rosner R Rimane E Vogel A Rau J Hagl M . Treating prolonged grief disorder with prolonged grief-specific cognitive behavioral therapy: study protocol for a randomized controlled trial. Trials. (2018) 19:241. doi: 10.1186/s13063-018-2618-3 29678193 PMC5910599

[82] van Dis EAM van Veen SC Hagenaars MA Batelaan NM Bockting CLH van den Heuvel RM . Long-term outcomes of cognitive behavioral therapy for anxiety-related disorders: A systematic review and meta-analysis. JAMA Psychiatry. (2020) 77:265–73. doi: 10.1001/jamapsychiatry.2019.3986 PMC6902232 31758858

[83] Mann JJ Apter A Bertolote J Beautrais A Currier D Haas A . Suicide prevention strategies: a systematic review. Jama. (2005) 294:2064–74. doi: 10.1001/jama.294.16.2064 16249421

[84] Zalsman G Hawton K Wasserman D van Heeringen K Arensman E Sarchiapone M . Suicide prevention strategies revisited: 10-year systematic review. Lancet Psychiatry. (2016) 3:646–59. doi: 10.1016/S2215-0366(16)30030-X 27289303

[85] Patel V Gonsalves PP . Suicide prevention: Putting the person at the center. PloS Med. (2019) 16:e1002938. doi: 10.1371/journal.pmed.1002938 31568477 PMC6768450

