# Supplementary figures and images for: Suicidal incidence and gender-based discrepancies in prolonged grief disorder: insights from a meta-analysis and systematic review
Source: Front Psychiatry. 2024 Aug 15;15:1427486. doi: 10.3389/fpsyt.2024.1427486 (PMC11358064; doi:10.3389/fpsyt.2024.1427486)

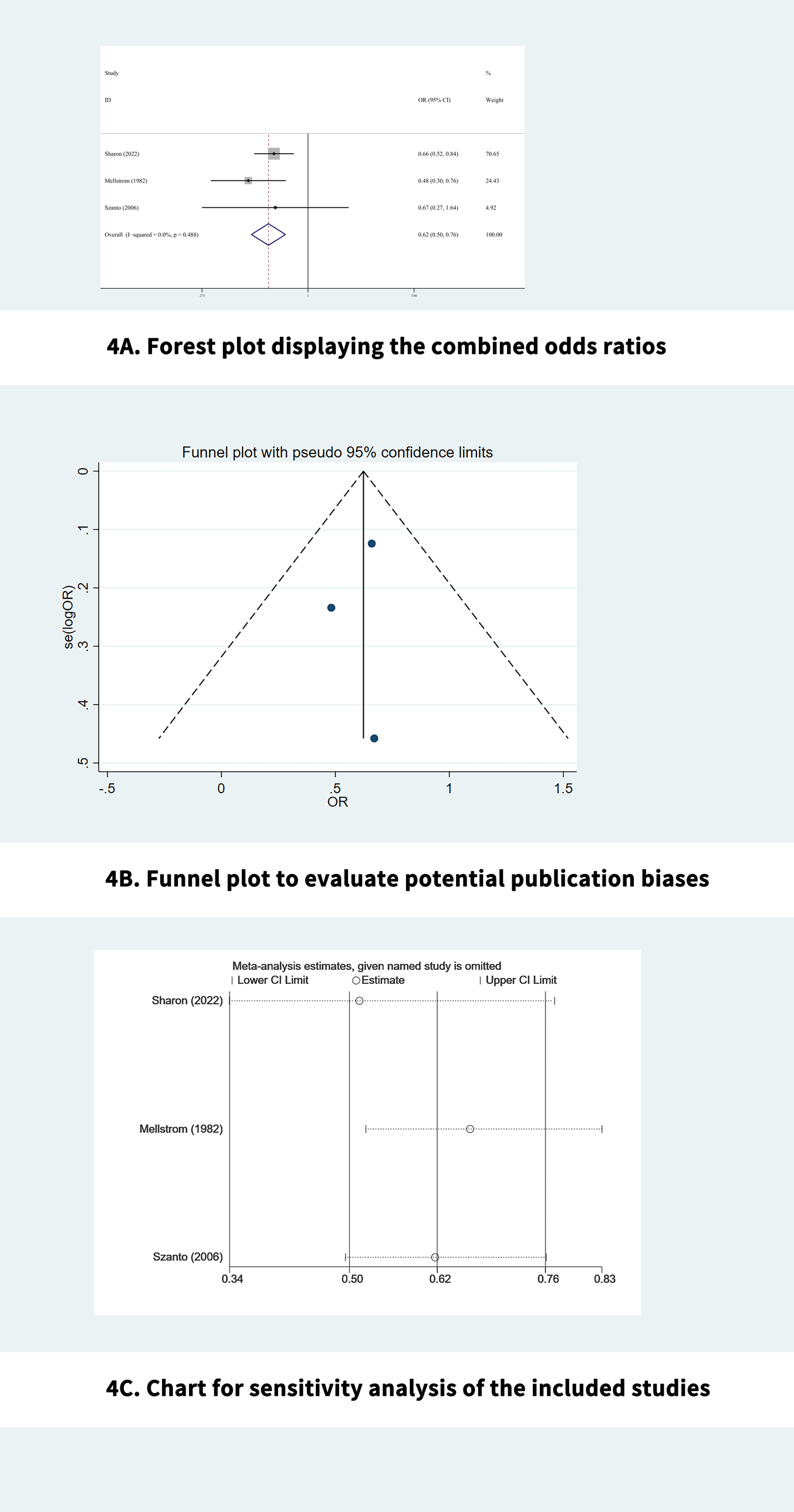

Supplement: Supplementary Figure 4 — Meta-analysis of self-injury: (A) Forest plot displaying the combined odds ratios; (B) Funnel plot to evaluate potential publication biases; (C) Chart for sensitivity analysis of the included studies. [file Image4.tif]
