# Supplementary material for: Suicidal incidence and gender-based discrepancies in prolonged grief disorder: insights from a meta-analysis and systematic review
Source: Front Psychiatry. 2024 Aug 15;15:1427486. doi: 10.3389/fpsyt.2024.1427486 (PMC11358064; doi:10.3389/fpsyt.2024.1427486)
Supplement: Supplementary file 5 [file Table1.docx]

**Supplementary Table 1. Database search strategy**

1. **pubmed Database search strategy**

| Search  number | Query | Results |
| --- | --- | --- |
| #10 | #3 and #6 and #9 | 64 |
| #9 | #8 or #9 | 2,472,871 |
| #8 | (((((((((((((((((((((((((((((((Sex Characteristics[Title/Abstract]) OR (gender difference[Title/Abstract])) OR (Genders[Title/Abstract])) OR (Male[Title/Abstract])) OR (Female[Title/Abstract])) OR (Man[Title/Abstract])) OR (Woman[Title/Abstract])) OR (Boy[Title/Abstract])) OR (girl[Title/Abstract])) OR (Characteristic, Sex[Title/Abstract])) OR (Sex Characteristic[Title/Abstract])) OR (Sexual Dimorphism[Title/Abstract])) OR (Dimorphism, Sexual[Title/Abstract])) OR (Sexual Dimorphisms[Title/Abstract])) OR (Gender Differences[Title/Abstract])) OR (Gender Difference[Title/Abstract])) OR (Sex Dimorphism[Title/Abstract])) OR (Dimorphism, Sex[Title/Abstract])) OR (Sex Dimorphisms[Title/Abstract])) OR (Gender Characteristics[Title/Abstract])) OR (Characteristic, Gender[Title/Abstract])) OR (Gender Characteristic[Title/Abstract])) OR (Gender Dimorphism[Title/Abstract])) OR (Dimorphism, Gender[Title/Abstract])) OR (Gender Dimorphisms[Title/Abstract])) OR (Sex Differences[Title/Abstract])) OR (Sex Differences Difference, Sex[Title/Abstract])) OR (Sex Difference[Title/Abstract])) OR (Sexual Dichromatism[Title/Abstract])) OR (Dichromatism, Sexual[Title/Abstract])) OR (Dichromatisms, Sexual[Title/Abstract])) OR (Sexual Dichromatisms[Title/Abstract]) | 2,452,114 |
| #7 | "Sex Characteristics"[Mesh] | 62,296 |
| #6 | #4 or #5 | 112,645 |
| #5 | ((((((((((((Suicide[Title/Abstract]) OR (Suicidal Ideation[Title/Abstract])) OR (Suicide Prevention[Title/Abstract])) OR (Suicide, Assisted[Title/Abstract])) OR (Suicide, Attempted[Title/Abstract])) OR (Suicide, Completed[Title/Abstract])) OR (suicide attempt[Title/Abstract])) OR (Suicide mortality rate[Title/Abstract])) OR (self-injury[Title/Abstract])) OR (suicide program[Title/Abstract])) OR (Non-suicidal[Title/Abstract])) OR (suicidal behavior[Title/Abstract])) OR (self-injury behavior[Title/Abstract]) | 89,827 |
| #4 | "Suicide"[Mesh] | 76,855 |
| #3 | #1 or #2 | 15,091 |
| #2 | **((((((((((((((Disorder, Prolonged Grief[Title/Abstract]) OR (Grief Disorder, Prolonged[Title/Abstract])) OR (Prolonged Grief Disorders[Title/Abstract])) OR (Prolonged Grief[Title/Abstract])) OR (PGD[Title/Abstract])) OR (complicated grief[Title/Abstract])) OR (Persistent Complex Bereavement Disorder[Title/Abstract])) OR (Persistent Complex Grief Disorder[Title/Abstract])) OR (traumatic grief[Title/Abstract])) OR (Pathological grief[Title/Abstract])) OR (Complex sadness[Title/Abstract])) OR (acute grief[Title/Abstract])) OR (Abnormal sadness[Title/Abstract])) OR (Grief[Title/Abstract])) OR (Mourn[Title/Abstract])** | 15,091 |
| #1 | "Prolonged Grief Disorder"[Mesh] | 92 |

Date Run: 2023/11/24

# Web of science Database search strategy

| Search  number | Query | Results |
| --- | --- | --- |
| #4 | #1 AND #2 AND #3 and Preprint Citation Index (Exclude – Database) | 1,066 |
| #3 | Sex Characteristics (Topic) or gender difference (Topic) or Genders (Topic) or Male (Topic) or Female (Topic) or Man (Topic) or Woman (Topic) or Boy (Topic) or girl (Topic) or Characteristic, Sex (Topic) or Sex Characteristic (Topic) or Sexual Dimorphism (Topic) or Dimorphism, Sexual (Topic) and Sexual Dimorphisms (Topic) or Gender Differences (Topic) or Gender Difference (Topic) or Sex Dimorphism (Topic) or Dimorphism, Sex (Topic) and Sex Dimorphisms (Topic) or Gender Characteristics (Topic) or Characteristic, Gender (Topic) or Gender Characteristic (Topic) or Gender Dimorphism (Topic) or Dimorphism, Gender (Topic) or Gender Dimorphisms (Topic) or Sex Differences (Topic) or Difference, Sex (Topic) or Sex Difference (Topic) or Dichromatism, Sexual (Topic) or Sexual Dichromatism (Topic) or Sexual Dichromatisms (Topic) and Preprint Citation Index (Exclude – Database) | 17,983,099 |
| #2 | Suicide (Topic) or Suicidal Ideation (Topic) or Suicide Prevention (Topic) or Suicide, Assisted (Topic) or Suicide, Attempted (Topic) or Suicide, Completed (Topic) or suicide attempt (Topic) or Suicide mortality rate (Topic) or self-injury (Topic) or suicide program (Topic) or Non-suicidal (Topic) or suicidal behavior (Topic) or self-injury behavior (Topic) and Preprint Citation Index (Exclude – Database) | 169,509 |
| #1 | Prolonged Grief Disorder (Topic) or Disorder, Prolonged Grief (Topic) or Grief Disorder, Prolonged (Topic) or Prolonged Grief Disorders (Topic) or Prolonged Grief (Topic) or PGD (Topic) or complicated grief (Topic) or Persistent Complex Bereavement Disorder (Topic) or Persistent Complex Grief Disorder (Topic) or traumatic grief (Topic) or Pathological grief (Topic) or Complex sadness (Topic) or acute grief (Topic) or Abnormal sadness (Topic) or Grief (Topic) or Mourn (Topic) and Preprint Citation Index (Exclude – Database) | 42,295 |

Date Run: 2023/11/24

# Embase Database search strategy

| No. | Query | Results |
| --- | --- | --- |
| #4 | #1 AND #2 AND #3 | 89 |
| #3 | 'sex characteristics':ab,ti OR genders:ab,ti OR male:ab,ti OR female:ab,ti OR boy:ab,ti OR woman:ab,ti OR man:ab,ti OR girl:ab,ti OR 'characteristic, sex':ab,ti OR 'sex characteristic':ab,ti OR 'sexual dimorphism':ab,ti OR 'dimorphism, sexual':ab,ti OR 'sexual dimorphisms':ab,ti OR 'gender difference':ab,ti OR 'gender differences':ab,ti OR 'sex dimorphism':ab,ti OR 'dimorphism, sex':ab,ti OR 'sex dimorphisms':ab,ti OR 'gender characteristics':ab,ti OR 'characteristic, gender':ab,ti OR 'gender characteristic':ab,ti OR 'dimorphism, gender':ab,ti OR 'gender dimorphisms':ab,ti OR 'sex differences':ab,ti OR 'difference, sex':ab,ti OR 'sexual dichromatism':ab,ti OR 'dichromatism, sexual':ab,ti OR 'sex difference':ab,ti OR 'sexual dichromatisms':ab,ti | 3,637,285 |
| #2 | suicide:ab,ti OR 'suicide, assisted':ab,ti OR 'suicide prevention':ab,ti OR 'suicidal ideation':ab,ti OR 'suicide, attempted':ab,ti OR 'suicide, completed':ab,ti OR 'suicide attempt':ab,ti OR 'suicide mortality rate':ab,ti OR 'self injury':ab,ti OR 'suicide program':ab,ti OR 'suicidal behavior':ab,ti OR 'self-injury behavior':ab,ti OR 'non suicidal':ab,ti | 111,355 |
| #1 | 'prolonged grief disorder':ab,ti OR 'disorder, prolonged grief':ab,ti OR 'grief disorder, prolonged':ab,ti OR 'prolonged grief disorders':ab,ti OR 'prolonged grief':ab,ti OR pgd:ab,ti OR 'complicated grief':ab,ti OR 'persistent complex bereavement disorder':ab,ti OR 'pathological grief':ab,ti OR 'persistent complex grief disorder':ab,ti OR 'traumatic grief':ab,ti OR 'complex sadness':ab,ti OR 'acute grief':ab,ti OR 'abnormal sadness':ab,ti OR grief:ab,ti OR mourn:ab,ti | 22,248 |

Date Run: 2023/11/24

# Cochrane Database search strategy

| ID | Search | Hits |
| --- | --- | --- |
| #7 | (#1 OR #2) AND (#3 OR #4) AND (#5 OR #6) | 38 |
| #6 | MeSH descriptor: [Sex Characteristics] explode all trees | 1,652 |
| #5 | Sex Characteristics OR gender difference OR Genders OR Male OR Female OR Man OR Woman OR Boy OR girl OR Characteristic, Sex OR Sex Characteristic OR Sexual Dimorphism OR Dimorphism, Sexual OR Sexual Dimorphisms OR Gender Differences OR Gender Difference OR Sex Dimorphism OR Dimorphism, Sex OR Sex Dimorphisms OR Gender Characteristics OR Characteristic, Gender OR Gender Characteristic OR Gender DimorphismOR Dimorphism, Gender OR Gender Dimorphisms OR Sex Differences OR Difference, Sex OR Sex Difference OR Sexual Dichromatism OR Dichromatism, Sexual OR Dichromatisms, Sexual OR Sexual Dichromatisms1156771 | 1,156,771 |
| #4 | MeSH descriptor: [Suicide] explode all trees | 2,129 |
| #3 | (Suicidal Ideation) OR (Suicide Prevention) OR (Suicide, Assisted) OR (Suicide, Attempted) OR (Suicide, Completed) OR (Suicide) OR (suicide attempt) OR (Suicide mortality rate) OR (self-injury) OR (suicide program) OR (Non-suicidal self-injury) OR (suicidal behavior) OR (self-injury behavior) | 7,786 |
| #2 | MeSH descriptor: [Prolonged Grief Disorder] explode all trees | 8 |
| #1 | Prolonged Grief Disorder OR Disorder, Prolonged Grief OR Grief Disorder, Prolonged OR Prolonged Grief Disorders OR Prolonged Grief OR PGD OR complicated grief OR Persistent Complex Bereavement Disorder OR Persistent Complex Grief Disorder OR traumatic grief OR Pathological grief OR Complex sadness OR acute grief OR Abnormal sadness OR Grief OR Mourn | 1,243 |

Date Run: 2023/11/24

# PsycINFO Database search strategy

| ID | Search | Hits |
| --- | --- | --- |
| #4 | #1 AND #2 AND #3 | 226 |
| #3 | TX Sex Characteristics OR TX gender difference OR TX Genders OR TX Male OR TX Female OR TX Man OR TX Woman OR TX Boy OR TX girl OR TX Characteristic, Sex OR TX Sex Characteristic OR TX Sexual Dimorphism OR TX Dimorphism, Sexual OR TX Sexual Dimorphisms OR TX Gender Differences OR TX Gender Difference OR TX Sex Dimorphism OR TX Dimorphism, Sex OR TX Sex Dimorphisms OR TX Gender Characteristics OR TX Characteristic, Gender OR TX Gender Characteristic OR TX Gender DimorphismOR TX Dimorphism, Gender OR TX Gender Dimorphisms OR TX Sex Differences OR TX Difference, Sex OR TX Sex Difference OR TX Sexual Dichromatism OR TX Dichromatism, Sexual OR TX Dichromatisms, Sexual OR TX Sexual Dichromatisms | 2,128,343 |
| #2 | TX Suicidal Ideation OR TX Suicide Prevention OR TX Suicide, Assisted OR TX Suicide, Attempted OR TX Suicide, Completed OR TX Suicide OR TX suicide attempt OR TX Suicide mortality rate OR TX self-injury OR TX suicide program OR TX Non-suicidal OR TX suicidal behavior OR TX self-injury behavior | 65,564 |
| #1 | TX Prolonged Grief Disorder OR TX Disorder, Prolonged Grief OR TX Grief Disorder, Prolonged OR TX Prolonged Grief Disorders OR TX Prolonged Grief OR TX PGD OR TX complicated grief OR TX Persistent Complex Bereavement Disorder OR TX Persistent Complex Grief Disorder OR TX traumatic grief OR TX Pathological grief OR TX Complex sadness OR TX acute grief OR TX Abnormal sadness OR TX Grief OR TX Mourn | 3,039 |

Date Run: 2023/11/24
