# Supplementary material for: Suicidal incidence and gender-based discrepancies in prolonged grief disorder: insights from a meta-analysis and systematic review
Source: Front Psychiatry. 2024 Aug 15;15:1427486. doi: 10.3389/fpsyt.2024.1427486 (PMC11358064; doi:10.3389/fpsyt.2024.1427486)
Supplement: Supplementary file 6 [file Table2.docx]

| Study | Selection |  |  |  | Comparability | Exposure |  |  | Scores |
| --- | --- | --- | --- | --- | --- | --- | --- | --- | --- |
|  | Adequate definition of cases | Representat iveness of the cases | Selection of con trols | Definition of con trols | Control for important factor | Ascertain-ment of exposure | Same method of ascertain-ment for cases and contrels | Non-response rate |  |
| Sharon et al.,2022 | ☆ |  |  | ☆ | ☆☆ | ☆ | ☆ |  | 6 |
| Mellstrom et al.,1982 | ☆ | ☆ | ☆ | ☆ | ☆☆ | ☆ | ☆ |  | 8 |
| Szanto et al.,2006 | ☆ | ☆ | ☆ | ☆ | ☆☆ | ☆ | ☆ |  | 8 |
| Ann M et al.,2005 | ☆ | ☆ | ☆ | ☆ | ☆☆ |  | ☆ |  | 7 |
| Song et al.,2015 | ☆ | ☆ | ☆ | ☆ | ☆☆ | ☆ |  |  | 7 |
| Katalin et al.,2010 | ☆ | ☆ | ☆ | ☆ | ☆☆ |  | ☆ |  | 7 |
| Latham et al.,2004 | ☆ | ☆ | ☆ | ☆ | ☆ | ☆ | ☆ |  | 7 |
| [Grafiadeli](https://pubmed.ncbi.nlm.nih.gov/?sort=date&term=Grafiadeli+R&cauthor_id=34673315) et al.,2021 | ☆ | ☆ | ☆ | ☆ | ☆☆ | ☆ |  |  | 7 |
| Abbott et al.,2014 | ☆ | ☆ | ☆ | ☆ | ☆☆ | ☆ | ☆ |  | 8 |
| Shilubane et al.,2013 | ☆ | ☆ | ☆ | ☆ | ☆ | ☆ | ☆ |  | 7 |
| Judy et al.,2020 | ☆ | ☆ | ☆ | ☆ | ☆ | ☆ | ☆ | ☆ | 8 |
| Williams et al.,2018 | ☆ |  |  | ☆ | ☆☆ | ☆ | ☆ |  | 6 |
| Hill et al.,1969 | ☆ | ☆ |  | ☆ | ☆ | ☆ |  | ☆ | 6 |
| Wilcox et al.,2015 | ☆ | ☆ | ☆ | ☆ | ☆☆ | ☆ | ☆ |  | 8 |
| Sibold et al.,2015 | ☆ | ☆ | ☆ | ☆ | ☆☆ | ☆ |  |  | 7 |
| Choi et al.,2017 | ☆ | ☆ | ☆ | ☆ | ☆☆ | ☆ |  |  | 7 |
| Mikael et al., 2014 | ☆ | ☆ | ☆ | ☆ | ☆☆ | ☆ | ☆ |  | 8 |
| Bottomley et al.,2022 | ☆ | ☆ | ☆ | ☆ | ☆☆ | ☆ |  |  | 7 |
| Burrell et al.,2018 | ☆ | ☆ | ☆ | ☆ | ☆☆ | ☆ | ☆ |  | 8 |
| Helsing et al.,1982 | ☆ | ☆ | ☆ | ☆ | ☆ | ☆ | ☆ |  | 7 |
| Mikael et al.,2013 | ☆ | ☆ | ☆ | ☆ | ☆☆ | ☆ | ☆ |  | 8 |

Supplementary Table 2. Detailed Classification and Individual Scores for Selected Studies
